# Supplementary material for: Identification of photoperiod-induced specific miRNAs in the adrenal glands of Sunite sheep (Ovis aries)
Source: Front Vet Sci. 2022 Jul 22;9:888207. doi: 10.3389/fvets.2022.888207 (PMC9354845; doi:10.3389/fvets.2022.888207)
Supplement: Supplementary file 1 [file Data_Sheet_1.ZIP › Supplementary file/miRDeep2 file.zip!/miRDeep2 file/Novel_Structure/SPLP42A1/Novel_405.pdf]

Provisional ID	:	NC_019484.2_31512
Score total	:	1.4
Score for star read(s)	:	-1.3
Score for read counts	:	0
Score for mfe	:	1.1
Score for randfold	:	1.6
Score for cons. seed	:	
Total read count	:	18
Mature read count	:	18
Loop read count	:	0
Star read count	:	0

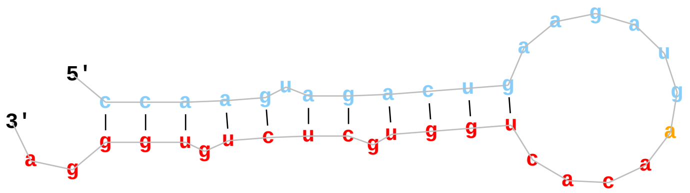

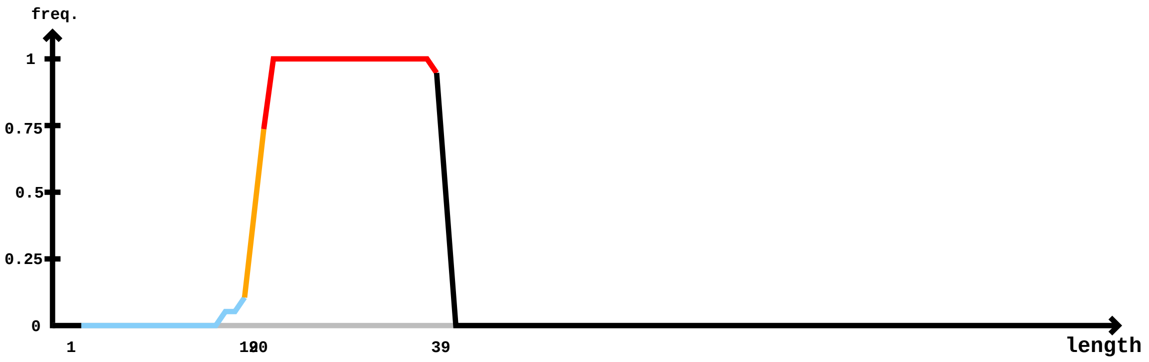


```
Star          Mature
5'- cccaaguagacugaagaugaacacuggugcucugugggaugagguaguagauuguauaguuuuagggucauaccccaucuuggagauaacuauacagucuacugucuuu   -3'   exp
    ((((((.((((((...........)))).)))).))))..((((((((((((((((((((((((((((........))))))))....)))))))))))))))))))))).        reads   mm   sample
    ................augaacacuggugcucuguggga......................................................................        1       0    seq
    ..................gaacacuggugcucuguggga......................................................................        1       0    seq
    ...................aacacuggugcucuguggga......................................................................        4       0    seq
    ....................acacuggugcucuguggga......................................................................        8       0    seq
    .....................cacuggugcucugugggg.......................................................................        1       0    seq
    .....................cacuggugcucuguggga......................................................................        4       0    seq
```
